# The Impact of the Reduction in Environmental Pollution during COVID-19 Lockdown on Healthy Individuals

**DOI:** 10.3390/toxics12070492

**Published:** 2024-07-04

**Authors:** Christian Romero-Mesones, Miquel de Homdedeu, David Soler-Segovia, Carlos Gómez-Ollés, David Espejo-Castellanos, Inigo Ojanguren, Berta Saez-Gimenez, María-Jesús Cruz, Xavier Munoz

**Affiliations:** 1 Pulmonology Service, Hospital Universitari Vall d’Hebron, 08035 Barcelona, Spain; christian.romero@vhir.org (C.R.-M.); mhomdedeu@bst.cat (M.d.H.); david.soler@vhir.org (D.S.-S.); carlos.gomez@vallhebron.cat (C.G.-O.); david.espejo@vhir.org (D.E.-C.); inigo.ojanguren@vallhebron.cat (I.O.); berta.saez@vallhebron.cat (B.S.-G.); xavier.munoz@vallhebron.cat (X.M.); 2 CIBER Enfermedades Respiratorias (CibeRes), 28029 Madrid, Spain; 3 Medicine Department, Universitat Autònoma de Barcelona, 08193 Barcelona, Spain; 4 Department of Cell Biology, Physiology and Immunology, Universitat Autònoma de Barcelona, 08193 Barcelona, Spain

**Keywords:** air pollutants, oxidative stress, cytokines, eosinophils

## Abstract

The lockdown imposed to combat the COVID-19 pandemic produced a historic fall in air pollution in cities like Barcelona. This exceptional situation offered a unique context in which to examine the effects of air pollutants on human health. The present study aims to determine and compare the oxidative stress biomarkers Th1/Th2 and inflammatory-related cytokines in healthy individuals first during lockdown and then six months after the easing of the restrictions on mobility. A prospective study of a representative sample of 58 healthy, non-smoking adults was carried out. During lockdown and six months post-easing of restrictions, blood samples were drawn to measure the percentage of eosinophils, levels of Th1/Th2 and inflammatory-related cytokines assessed by a multiplex assay (BioRad Laboratories S.A., Marnes-la-Coquette, France), and levels of 8-isoprostane, glutathione peroxidase activity, and myeloperoxidase (Cayman Chemical Co., Ann Arbor, MI, USA), to assess their value as biomarkers of oxidative stress. Six months after easing mobility restrictions, increases in the levels of 8-isoprostane (*p* < 0.0001), IL-1β (*p* = 0.0013), IL-1ra (*p* = 0.0110), IL-4 (*p* < 0.0001), IL-13 (*p* < 0.0001), G-CSF (*p* = 0.0007), and CCL3 (*p* < 0.0001) were recorded, along with reductions in glutathione peroxidase (*p* < 0.0001), IFN-γ (*p* = 0.0145), TNFα (*p* < 0.0001), IP-10 (*p* < 0.0001), IL-2 (*p* < 0.0001), IL-7 (*p* < 0.0001), basic FGF (*p* < 0.0001), CCL4 (*p* < 0.0001), and CCL5 (*p* < 0.0001). No significant differences were observed in the rest of the biomarkers analyzed. The reduction in environmental pollution during the COVID-19 lockdown significantly lowered the levels of oxidative stress, systemic inflammation, and Th2-related cytokines in healthy people.

## 1. Introduction

Air pollution is a major public health problem that affects a large part of the world’s population [1]. The impacts of air pollution on human health are wide-ranging, the most common being the aggravation of respiratory and cardiovascular diseases [2]. High levels of particulate matter (PM), ozone, sulfur dioxide, and nitrous oxide (O_3_, SO_2_, and NO_2_) can precipitate the appearance of symptoms, increasing the number of visits to emergency services and hospitalizations due to decompensation of the disease [3]. Various studies have shown that exposure to pollutants can cause lung and systemic inflammation, changes in the immune response, increases in oxidative stress, and decreased lung function in people with previous respiratory pathologies [4,5,6,7]. These effects have also been observed in healthy individuals exposed to pollutants; however, studies in this regard are scarce, and have focused mainly on children and the elderly, who are more susceptible to the effects of inhaling toxic substances [8,9].

The confinement imposed to combat the COVID-19 pandemic produced a historic fall in air pollution in cities such as Barcelona [10]. Between 13 March and 21 June 2020, lockdown measures were introduced in Spain to reduce the COVID-19 epidemic curve, restricting social contact, vehicular transport and economic activity. Levels of PM_2.5_ and NO_2_ began to fall dramatically in the city as of 13 March, coinciding with the adoption of the first confinement measures. During lockdown, NO_2_ levels were four times lower than the levels that the WHO considers harmful to health (40 micrograms per cubic meter) and which in normal times are systematically exceeded in Barcelona [11]. This exceptional situation created a unique context for studying the effects of a sudden reduction in contamination on the immune system of healthy individuals. For all the above reasons, the present study aimed to determine and compare the oxidative stress biomarkers, Th1/Th2, and inflammatory-related cytokines in healthy individuals during lockdown and six months after the easing of mobility restrictions.

## 2. Material and Methods

### 2.1. Study Design and Subjects

Prospective observational study with a repeated measures design in which environmental pollutants were the independent variables and oxidative stress and immunological biomarkers were the primary outcome variables.

Fifty-eight healthy individuals recruited from hospital staff participated in this study. Exclusion criteria were current or past smoking habits, current pulmonary, cardiovascular, liver or renal disease, or residence outside the metropolitan area of Barcelona. None of the participants were taking any medication. Blood samples were acquired twice in each individual: after three months of lockdown (Low Air Pollution levels: LAP) and six months after the easing of mobility restrictions in Barcelona (High Air Pollution levels: HAP). Whole venous peripheral blood samples were drawn at the same time at each visit (around 8:00 a.m.). For each individual and visit, blood was collected into two BD Vacutainer K2E (EDTA) tubes (Becton Dickinson, Berkshire, UK) and a single BD SST™ II Advance tube (Becton Dickinson, UK). One EDTA tube was used to assess the hematological profile, and the other was immediately centrifuged (1000× *g*, 10 min, 4 °C) to obtain plasma. After clot formation, plasma was separated by centrifuging SST tubes (2000× *g*, 15 min, 4 °C). Plasma samples were collected and stored at −80 °C until analysis. Blood eosinophil counts were measured in fresh blood samples and reported in %. Atopy was determined using ImmunoCAP Phadiatop (Phadia AB, Uppsala, Sweden) containing inhalant allergens of cat, dog, horse, birch, timothy, mugwort, *Dermatophagoides pteronyssinus*, and Cladosporium sp. Samples that exceeded 0.35 kUA/L were considered positive.

This study was approved by the Ethics Committee of Hospital Universitari Vall d’Hebron (PR(AG)251/2020). All participants provided written informed consent.

### 2.2. Oxidative Stress Biomarker Measurement

Levels of 8-isoprostane, glutathione peroxidase activity, and myeloperoxidase (Cayman Chemical Co., Ann Arbor, MI, USA) were assessed according to the manufacturer’s instructions.

### 2.3. Cytokine Measurement

Levels of IL1β, IL1ra, IL-2, IL-4, IL-5, IL-6, IL-7, IL-8, IL-10, IFN-γ, IP-10, TNF-α, G-CSF, basic FGF, CCL2, CCL3, CCL4, and CCL5 were measured in serum samples by a cytokine magnetic bead panel (Bio-Plex Pro Human Cytokine 27-plex Assay, BioRad Laboratories S.A., Marnes-la-Coquette, France) according to the manufacturer’s instructions.

### 2.4. Data Analysis

Data are shown as means and standard deviations (SDs) or as individual data and group medians. Parametric and nonparametric statistical tests were performed according to data distribution, as determined by the Shapiro–Wilk normality test. Comparisons were performed using the Kruskal–Wallis test followed by Dunn’s multiple comparisons test and one-way ANOVA followed by Tukey’s post hoc test. A level of *p* < 0.05 was considered to be significant. Statistical analysis was performed with SPSS statistical software system version 20 (SPSS, Inc., Chicago, IL, USA) and graphs were generated using GraphPad software (GraphPad Prism 6.01, GraphPad Software Inc., San Diego, CA, USA).

## 3. Results

### 3.1. Demographic Characteristics of the Study Population

The participants comprised 58 subjects (22 males). The median age of the study population was 34 (range 22–62) years. Thirty-one (53%) subjects were atopic. No significant differences were observed in the % of eosinophils between the two study periods: Median 2.7 (range: 0.4–9.3) and 2.3 (0.5–11.2) in the LAP and HAP groups, respectively (*p* = 0.07). Thirty-six (62%) lived in the city of Barcelona and 22 (38%) in the metropolitan area.

### 3.2. Oxidative Stress Levels

**Figure 1:**
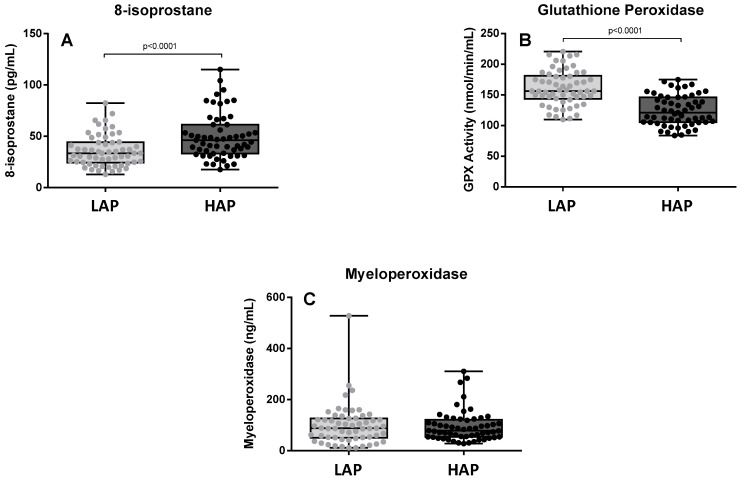
Levels of 8-isoprostane (**A**), glutathione peroxidase activity (**B**), and myeloperoxidase (**C**) in the two study periods. LAP: Low Air Pollution levels; HAP: High Air Pollution levels. Data expressed as medians (range). 8-isoprostane: LAP: 33.56 pg/mL (12.96–82.38); HAP: 46.31 pg/mL (17.74–115.2). Glutathione peroxidase: LAP: 156.4 nmol/min/mL (109.8–220.7); HAP: 121 nmol/min/mL (83.89–175). Myeloperoxidase: LAP: 87.65 ng/mL (11.1–528.1); 78.67 ng/mL (27.62–310.8).

Figure 1 shows the levels of 8-isoprostane, glutathione peroxidase activity, and myeloperoxidase at both assessment points. 8-isoprostane levels were significantly higher in the HAP period than in the LAP period (*p* < 0.0001) (Figure 1A). A decrease in glutathione peroxidase activity was observed in the HAP period (*p* < 0.0001) (Figure 1B). No significant differences were observed in the levels of myeloperoxidase between the two time points (Figure 1C).

### 3.3. Cytokine Levels

**Figure 2:**
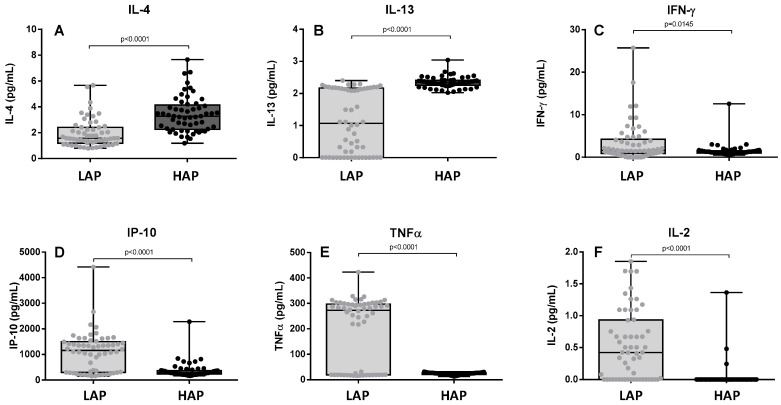
Levels of IL-4 (**A**), IL-13 (**B**), IFN-γ (**C**), IP-10 (**D**), TNF-α (**E**), and IL2 (**F**) in the two study periods. LAP: Low Air Pollution levels; HAP: High Air Pollution levels. Data expressed as median (range). IL-4: LAP: 1.575 pg/mL (0.7953–5.671); HAP: 3.283 pg/mL (1.185–7.664). IL-13: LAP: 1.068 pg/mL (0–2.403); HAP: 2.338 pg/mL (2.024–3.04). IFN-γ: LAP: 1.577 pg/mL (0–25.7); HAP: 1.197 pg/mL (0.4764–12.56). TNF-α: LAP: 272.9 pg/mL (15.75–423.2); HAP: 24.71 pg/mL (12.84–31.25). IL2: LAP: 0.4228 pg/mL (0–1.852); HAP: 0 pg/mL (0–1.364).

Figure 2 shows the levels of Th1/Th2 cytokines (IL-2, IL-4, IFN-γ, IP-10 and TNF-α). Levels of Th2 cytokines such as IL-4 and IL-13 were significantly higher in the HAP period than in the LAP period (*p* < 0.0001) (Figure 2A,B). In contrast, Th1 cytokine levels such as IFN-γ, IP-10, TNF-α, and IL-2 were lower in the HAP period (*p* = 0.0145, <0.0001, <0.0001 and <0.0001, respectively) (Figure 2C–F). IL-5 levels were only detectable in five patients.

**Figure 3:**
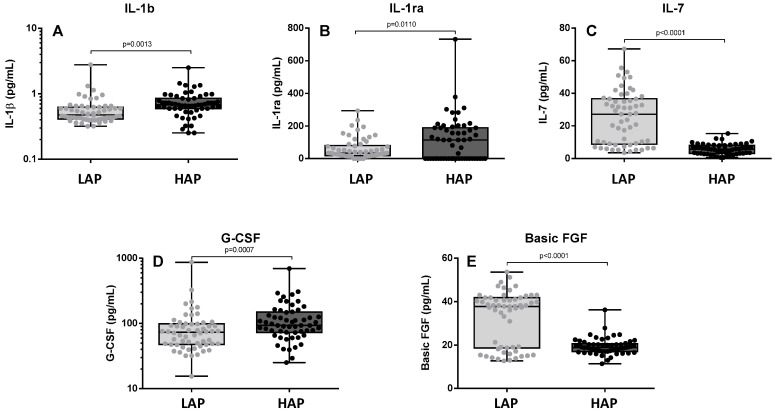
Levels of IL1β (**A**), IL1-ra (**B**), IL-7 (**C**), G-CSF (**D**), and basic FGF (**E**) in the two study periods. LAP: Low Air Pollution levels; HAP: High Air Pollution levels. Data expressed as median (range). IL1β: LAP: 0.4767 pg/mL (0.3195–2.786); HAP: 0.6777 pg/mL (0.2531–2.51). IL-1ra: LAP: 33.85 pg/mL (0–293.4); HAP: 114.4 pg/mL (0–732.2). IL-7: 27.16 pg/mL (3.502–67.2); HAP: 5.787 pg/mL (0.5594–15.33). G-CSF: 73.2 pg/mL (15.48–868.6); HAP: 93.29 pg/mL (25.04–695.6). Basic FGF: LAP: 37.85 pg/mL (12.71–53.69); HAP: 18.85 pg/mL (11.42–36.23).

Figure 3 shows the levels of pro-inflammatory and regulatory cytokines (IL-1β, IL-1ra, IL-7, G-CSF, and basic FGF). Levels of IL-1β, IL1ra, and G-CSF were significantly higher in the HAP period (*p* = 0.0013, 0.0110, and 0.0007, respectively) (Figure 3A–C). In contrast, levels of IL7 and basic FGF were lower in the HAP period (*p* < 0.0001) (Figure 3D,E). No significant differences were observed in the levels of IL-6 and IL-8; IL-10 levels were not detectable.

**Figure 4:**
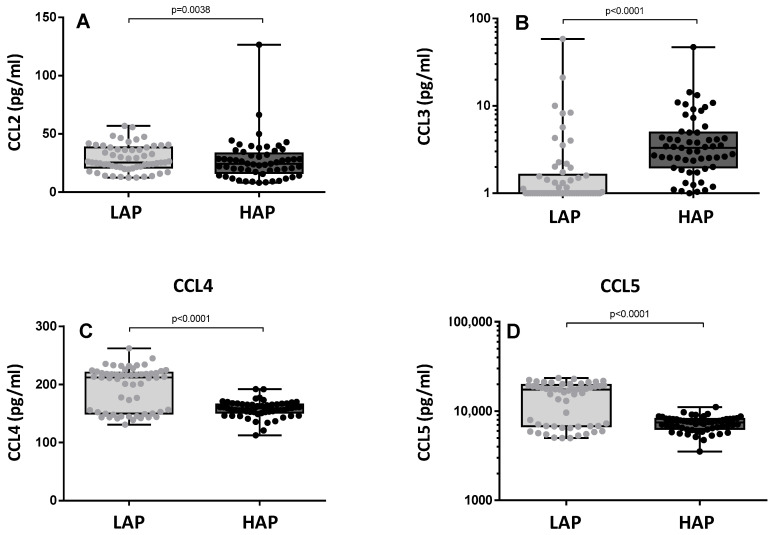
Levels of CC chemokines: CCL2 (**A**), CCL3 (**B**), CCL4 (**C**), and CCL5 (**D**) in the two study periods. LAP: Low Air Pollution levels; HAP: High Air Pollution levels. Data expressed as median (range). CCL2: LAP: 25.56 pg/mL (12.51–56.98); HAP: 24.48 pg/mL (8.18–126.6). CCL3: LAP: 0.7445 pg/mL (0.3322–58.22); HAP: 3.309 pg/mL (0.8479–47.07). CCL4: LAP: 212 pg/mL (130.8–262.4); HAP: 159.1 pg/mL (112.5–192.2). CCL5: LAP: 17486 pg/mL (4976–23,509); HAP: 7473 pg/mL (3521–11,093).

Figure 4 shows the levels of CC chemokines (CCL2, CCL3, CCL4, and CCL5). Levels of CCL3 were significantly higher in the HAP period (*p* < 0.0001) (Figure 4B), but levels of CCL4 and CCL5 were lower in the HAP period (*p* < 0.0001) (Figure 4C,D). No significant differences were observed in CCL2 (Figure 4A).

## 4. Discussion

The present study shows that the reduction in environmental pollution during the COVID-19 lockdown significantly lowered the levels of oxidative stress, systemic inflammation, and Th2-related cytokines in healthy people.

During the COVID-19 pandemic, a lockdown was imposed in many parts of the world with the aim of stopping the spread of the virus, which led to a dramatic reduction in activities that increase air pollution, especially road traffic. This situation made it possible to assess the impact of a massive switch-off of atmospheric pollutant sources and represented a unique opportunity to study the effects of pollution reduction on healthy people. Exposure to pollutants can have deleterious effects on the healthy population, producing lung inflammation and oxidative stress, as well as immunological changes and impairments in lung function [4,5,6]. However, most of the studies aimed at determining the effects of contamination in healthy populations are in vitro studies; clinical studies are scarce, and the majority of those carried out have sought to establish the effects of pollutants rather than the effects of reducing pollution. In this regard, the Global Burden of Disease Study has shown that significant health benefits can be achieved by reducing environmental exposure [12].

In fact, it is known that environmental pollutants can directly activate cell signaling pathways, and both cell culture and animal model studies have shown the profound effects of air pollutants on different cell types in the immune system [13]. It has been shown that exposure to pollutants can modify adaptive immune responses and induce high levels of pro-inflammatory cytokines as well as oxidative stress markers [13]. In this connection, among the most significant findings of the present study are the changes observed in oxidative stress biomarkers. During the period of high pollutant concentration, we observed elevated levels of 8-isoprostane, a prostaglandin (PG)-F2-like compound belonging to the F2 isoprostane class which is produced in vivo by the free radical-catalyzed peroxidation of arachidonic acid and is a biomarker of damage related to oxidative stress [14]. Several studies have found an association between 8-isoprostane levels and air pollution. Oxidative stress occurs due to an imbalance between the generation of reactive oxygen species (ROS) and endogenous antioxidant capacity, and is involved in the early stages of damage after exposure to pollutants [15]. Reactive oxygen species activate antioxidant defense mechanisms at low levels of oxidative stress, inflammatory responses at intermediate levels, and cytotoxic effects at high levels [15,16]. In parallel, in the period of high concentration of pollutants, we observed a decrease in levels of glutathione peroxidase, an antioxidant enzyme that plays a central role in the maintenance of the reducing cellular environment, which is required to maintain redox homeostasis. However, environmental pollutants may decrease glutathione peroxidase levels [17]; Kooter et al. [18] observed that acute exposure to diesel exhaust particles lowered glutathione peroxidase levels in the lung significantly, and in a murine model, Ribero et al. [19] found that exposure to diesel exhaust particles intensified changes in lung inflammatory and structural parameters and reduced glutathione peroxidase activity. Our findings corroborate these results in a real-life setting and suggest that polluted environments increase oxidative stress and reduce the effect of antioxidant mechanisms, even in healthy people. It should also be noted that these levels of oxidative stress are probably permanent in people who live in large cities.

The increase in oxidative stress induced by exposure to pollutants is also associated with the release of pro-inflammatory cytokines [19,20,21,22]. Some studies suggest that pro-inflammatory reactions tend to start with the release of early responding cytokines such as IL-1β that may subsequently regulate the expression of other cytokines and chemokines. However, secondary cytokines may also be activated more directly and independently by pollution through the activation of pro-inflammatory signaling pathways within the cells [23]. In the present study, an increase in the levels of IL-1β, IL-1ra, and G-CSF was observed in the HAP period, thus corroborating the data reported in experimental studies in a real-life setting. IL-1β is an important mediator of the inflammatory response [24] and G-CSF initiates the proliferation and differentiation of leukocytes into mature neutrophils [25]. In contrast, in the period of maximum concentration of pollutants, we observed decreased levels of IL-7, a pleiotropic cytokine essential for lymphocyte survival and expansion [26], and of basic FGF, which is involved in cell survival activities and tissue repair [27]. Decreases in the levels of CCL2, CCL4, and CCL5 and an increase in CCL3 were also observed in the present study. CC chemokines have been shown to play an important role in inflammation, as both CCL3 and CCL4 orchestrate the immune responses to infection or inflammation [28,29]. During inflammation, these two chemokines can contribute to the expression of several other pro-inflammatory cytokines including TNF-α, IL-1β, and IL-6 from activated macrophages and fibroblasts. In a cross-sectional molecular epidemiological study comparing workers exposed to diesel engine exhaust for around 20 years and a control group of unexposed workers, Dai et al. [30] found that exposure was associated with a significant decline in serum levels of CCL4 and that increased concentrations were associated with a strong fall in CCL3. Therefore, the falls in CCL2, CCL4, and CCL5 found in the HAP period in the present study may be the result of exposure to pollutants. Chemokines play an important role in eliciting an immune response by recruiting T cells, macrophages, and NK cells. These findings corroborate previous reports in the literature according to which exposure to environmental pollutants produces a chronic pro-inflammatory state.

In addition to the general pro-inflammatory nature of exposure to these pollutants, many studies suggest that air pollution increases Th2 immune responses [16]. In epidemiological studies and experimental models, exposure to environmental pollution has been identified as a potent source of immunomodulation. Pollution is known to affect several parameters of the allergic reaction [31]. It is responsible for the dysregulation of parameters specifically involved in allergy, namely IgE synthesis and the establishment of a type 2 cytokine profile [32]. Pollutants have also been shown to increase the potency of environmental allergy-causing proteins by inducing a greater IgE-mediated allergic response, as well as a more potent Th2 cytokine response and increased eosinophilic inflammation [13]. Environmental pollutants have also been shown to lower the expression of IL-2 and IFN-γ in T cells, resulting in a diminished Th1 cell immune response and the persistence of abnormally activated T cells [33]. In the present study, we observed increases in Th2 cytokines such as IL-4 and IL-13 and a decrease in Th1 cytokines such as IFN-γ, TNF-α, IL-2, and IP-10. In an allergic response based on allergic Th2 inflammation, interleukins IL-4, IL-5, and IL-13 are the main mediators [13]. IL-4 induces class switching from B cells to IgE, IL-5 is an eosinophil activator, and IL-13 stimulates B cell growth and differentiation and inhibits Th1 cells. For its part, one of the functions of IFN-γ is to direct the differentiation of CD4+ T lymphocytes into Th1 lymphocytes, and it has been suggested that IP-10 may have a role in the maintenance of protective, type 1-dominated responses in non-atopic subjects [34]. These results suggest that in polluted environments such as cities, the activation of Th2 lymphocytes is increased, thus predisposing inhabitants to an allergic response.

This study has some limitations. First of all, the population is restricted to the inhabitants in a large city; it would have been of interest to compare the results obtained with a rural population not continuously exposed to pollution. Second, the markers of oxidative stress and cytokines measured are not specific to the effect of the air pollutants, and other causative factors or confounders cannot be completely ruled out. Third, the population comprised hospital staff, as during lockdown it would have been unethical to ask a group of healthy people to come to the hospital to carry out this study. That said, hospital workers do not present any particular characteristics that differentiate them from other city dwellers. Fourth, the sample was small, again due to the difficulty of recruiting participants during lockdown. Fifth, the percentage of atopy is higher than that described for the general population, which stands at 30%. However, we did not observe differences in the response depending on whether or not the subjects in question presented atopy. Finally, in the present study, environmental pollution levels were not collected. These levels were based on the data collected in the air quality monitoring network in Barcelona [11]. In any case, these data show that the concentration of pollutants was drastically reduced during confinement.

## 5. Conclusions

In conclusion, the present study demonstrates that healthy people who live in a city habitually present increased levels of oxidative stress, systemic inflammation, and Th2 responses due to exposure to pollutants, and that these levels may fall when this exposure ceases. Our results suggest that the production of oxidative stress and the reduction in antioxidant defense mechanisms may be associated with the release of pro-inflammatory cytokines and chemokines, favoring a chronic pro-inflammatory state. Lastly, pollutants appear to increase the Th2 response, which may predispose to an allergic response. The results may indicate the ways in which policy measures introduced to reduce pollution in cities can have a direct impact on health.

## Data Availability

The data presented in this study are available on request from the corresponding author due to privacy reasons.
